# Systematic braiding of Smoke-Free Home SafeCare to address child maltreatment risk and secondhand smoke exposure: findings from a pilot study

**DOI:** 10.1186/s40814-023-01303-4

**Published:** 2023-05-12

**Authors:** Shannon Self-Brown, Elizabeth W. Perry, Manderley Recinos, Michaela A. Cotner, Kate Guastaferro, Shadé Owolabi, Claire A. Spears, Daniel J. Whitaker, Jidong Huang, Michelle C. Kegler

**Affiliations:** 1 Georgia State University — School of Public Health, 140 Decatur St. SE, Atlanta, GA 30303 USA; 2 New York University — School of Global Public Health, 708 Broadway, New York, NY 10003 USA; 3 Emory University — Rollins School of Public Health, 1518 Clifton Rd., Atlanta, GA 30322 USA

**Keywords:** Systematic braiding, Smoke-free home, Child maltreatment, Parenting, Child protection

## Abstract

**Background:**

Exposure to secondhand tobacco smoke (SHS) and child maltreatment are preventable threats to child health. Few evidence-based interventions target both SHS and child maltreatment risk. The purpose of this paper is to describe the systematic braiding process of two evidence-based programs to address child SHS in the home and maltreatment perpetration risk, and present results from the formative work and pilot study.

**Methods:**

The first 4 steps of the systematic braiding process were completed, including the following: (1) the identification of core elements of both programs, (2) the development of an initial draft of the braided curriculum (Smoke-Free Home SafeCare — SFH-SC), (3) an acceptability and feasibility pilot of SFH-SC with caregivers of young children who reported a smoker living in the home (*N* = 8), and (4) feedback collection on the braided curriculum from SafeCare Providers (*N* = 9).

**Results:**

Experts identified common pedagogical and theoretical underpinnings for the two programs and braided *Smoke-Free Homes: Some Things Are Better Outside* into two *SafeCare* modules. Caregiver feedback from the pilot demonstrated that participants were engaged with SFH-SC and felt supported and comfortable discussing SHS intervention content with the SFH-SC Provider. Caregiver self-reports indicated a slight increase in smoke-free home rules from baseline to follow-up and a notable reduction in parent stress on the Parent Stress Index of 5.9 points (*SD* = 10.2). SafeCare Provider feedback following intensive review of the curriculum indicated high feasibility for SFH-SC delivery.

**Conclusions:**

Parent and Provider findings suggest SFH-SC is a viable intervention that has potential to reduce the public health impact of SHS and child maltreatment for at-risk families.

**Protocol:**

The protocol for the pilot is not published elsewhere; however, the full protocol for the hybrid trial can be found here: https://clinicaltrials.gov/ct2/show/NCT05000632.

**Trial registration:**

NCT, NCT05000632. Registered 14 July 2021, there is not a separate registration number for the pilot.

**Supplementary Information:**

The online version contains supplementary material available at 10.1186/s40814-023-01303-4.

## Key messages regarding feasibility

- Uncertainties regarding the feasibility included the following: (1) Could SafeCare Providers be trained to effectively deliver a braided Smoke-Free Home SafeCare curriculum, and would they be invested in the content? (2) Would SafeCare families engage in discussions regarding creating a smoke-free home? (3) Were the inclusion/exclusion criteria set for families appropriate?
- The key feasibility findings are as follows: (1) SafeCare Providers were willing and capable to deliver the Smoke-Free Home SafeCare curriculum, (2) caregivers enrolled in and completed the braided curriculum, and (3) completers showed increases in smoke-free home rules and reductions in parent stress.
- Feasibility finding implications for the main study: The training and delivery approaches for Smoke-Free Home SafeCare were finalized and vetted, and based on the results, the training materials and protocol products were determined by the research team to be ready for implementation in the large Hybrid 1 trial. The study findings led the research team to make a change to the inclusion criteria related to smoking rules for caregiver participant enrollment.

## Background

### Child maltreatment risk and secondhand smoke exposure among young children

Young children living in under-resourced communities are disproportionately exposed to adverse childhood experiences that impact health [73, 23]. Specifically, children living in low-socioeconomic status (SES) households are at 3 to 7 times greater risk for being victims of maltreatment compared to children in higher-SES households [4, 47, 50, 52, 55]. Child maltreatment (i.e., physical, sexual, emotional abuse, and neglect) is one of the most impactful adverse experiences on young children’s health, socioemotional development, and life course. Child maltreatment can lead to damage to the physical and neural structure of the brain [8, 58, 69], mental health difficulties [25, 29, 38], and long-term physical health problems [7, 16, 18, 21, 44, 76].

Secondhand tobacco smoke (SHS) exposure is also disproportionately high among children living in low SES households [25, 35, 59]. The inverse relationship between SES and smoke exposure is well documented [14, 41, 48, 53]. SHS exposure is associated with numerous child health conditions, including but not limited to sudden infant death syndrome, respiratory and ear infections, asthma attacks, impaired lung growth, and childhood cancer [2, 70, 53, 76]. Higher rates of child behavioral problems have also been linked to SHS exposure in children [62, 83]. Smoke-free homes mitigate these outcomes by reducing SHS exposure for both nonsmokers and children [6, 27, 63, 72]. Notably, programs targeting SHS prevention are of greatest need among populations in which smoking bans are least common (e.g., households with low SES, one or two current smokers, parents with less than a college education, and single parents, [82]).

Child maltreatment and exposure to SHS are major, yet preventable threats to child health. Longitudinal studies have found beneficial effects of early intervention efforts for children living in resource-limited settings on long-term health and a range of social and psychological outcomes [5, 10, 19, 33, 34, 50, 72, 73]. Distinct, independent evidence-based interventions exist that reduce child maltreatment perpetration risk (e.g., [12, 13, 77] and SHS (e.g., [39]. However, to our knowledge, few evidence-based programs exist which jointly target maltreatment risk and SHS exposure.

### Intervention approaches for targeting two public health outcomes

There is an emerging literature on best practices for integrating two (or more) evidence-based programs to effectively and efficiently target multiple programmatic goals for children who are experiencing cumulative risk [15, 30]. One approach is to implement the interventions in a *parallel* manner such that a client receives concurrent full doses of both programs [15, 45]. Limitations to the parallel approach include greater time burden and decreased engagement for program participants. An alternative approach is to systematically braid the interventions by explicitly identifying the similarities and differences of the interventions’ conceptual theories, active practices in program delivery, and implementation procedures. Guastaferro et al. [30] standardized the method of systematic braiding and conducted a trial [31], in which two models with complementary foci were integrated while maintaining fidelity to the programs and implementation infrastructures to address child abuse outcomes among families with young children. In the best of circumstances, two evidence-based interventions can be combined to effectively address multiple health risks in populations of focus.

The current study focuses on the systematic braiding of two evidence-based interventions that target child maltreatment prevention and child SHS exposure in the home. For child maltreatment risk, the SafeCare® program was selected as the evidence-based intervention of focus. For child SHS exposure, the Smoke-Free Homes: Some Things Are Better Outside (SFH) program was selected as the evidence-based program of focus.

#### SafeCare

SafeCare is typically delivered in child protection service settings, although it is also delivered in high-risk prevention settings in some regions of the USA. SafeCare is a brief (18 weeks) home visitation program that is highly effective in reducing child maltreatment perpetration and improving behavioral outcomes for parents of young children (0 to 5 years) [12, 13, 77]. SafeCare includes 18 total sessions, divided into three 6-session modules, including the following: (1) parent–child interaction, a module that promotes positive parenting skills in daily routines and play activities,(2) home safety, which offers parent education on common household hazards and the securement and removal of hazards to prevent unintentional child injury,and (3) Child Health, a module that offers parental education on how to make effective health decisions when a young child is sick or injured. SafeCare is broadly disseminated across the USA and internationally. While SafeCare is primarily a program delivered via home visiting, since March 2020, the program has also been delivered virtually, with over 80% of SafeCare Providers across the USA and abroad delivering virtual sessions [67].

Multiple randomized controlled trials have demonstrated the positive impact of SafeCare with high-risk families, relative to case management services or to a no-treatment control, both in child welfare settings (i.e., after maltreatment has occurred) and in prevention settings (i.e., serving families at risk for maltreatment) [11–13, 46, 68]. In the largest published effectiveness study to date, a statewide comparative effectiveness trial of SafeCare in the Oklahoma child welfare system, SafeCare reduced child maltreatment recidivism by 26% relative to usual care [12, 13]. A recent study examining SafeCare effectiveness as compared to standard child welfare services across five states indicated that SafeCare participation was associated with favorable effects on parenting skills and parenting stress [77].

#### Smoke-Free Homes: Some Things are Better Outside (SFH)

SFH is a brief intervention that is effective in promoting adoption of smoke-free home rules among low-SES households [39, 56, 79]. SFH is designed to be a brief, 6-week intervention that is comprised of print materials and one 15–20 min coaching call, where participants are encouraged to follow five steps to creating a smoke-free home. Information on smoking cessation support (i.e., contact information for Quitline, United Way) is included in the caregiver packet,however, this is not a primary focus or outcome of the program. Three randomized control trials document significant SFH intervention effects, with 40.0 to 62.9% of clients reporting a smoke-free home at 6-month post-baseline [39, 56, 79]. The status of self-reported smoke-free homes was confirmed by air nicotine concentration at 3-month post-baseline. SFH was also shown to be effective in a dissemination trial conducted with five 2–1-1 agencies across multiple states [9]. The intervention is listed on National Cancer Institute’s list of evidence-based cancer prevention and control interventions [49] and was disseminated to grantees of the California’s Tobacco Control Program.

### Current study

Recent findings suggest there is a dire need for SHS prevention programs specifically for at-risk families as results from a SafeCare trial indicated that 61.3% (*n* = 174) of caregivers involved in interventions delivered through the child protection system reported daily smoking [61]. These findings offer further justification for the importance of addressing SHS exposure among families who are engaged in the SafeCare program. Thus, the systematic braiding of the SafeCare and smoke-free home programs has the potential to be a scalable approach to reduce cumulative risk for negative health outcomes among young children. In this paper, we will describe the first four steps of systematic braiding of Smoke-Free Home SafeCare (SFH-SC) and present results of the pilot study with caregivers of young children (step 3) as well as the detailed feedback on the braided curriculum from clinical program experts (step 4). Systematic braiding is a five-step process [30] that includes (1) cross-training in both curricula to identify common content and pedagogical approaches, (2) development of the initial braided curriculum with input from experts, (3) piloting the braided curriculum in an acceptability and feasibility pilot with end users, (4) modifications and additional piloting as necessary, and (5) wide implementation of the braided curriculum. The findings described herein inform the procedures to test SFH-SC in a large NCI-funded Hybrid trial type 1 that will inform future implementation (step 5) and provide a roadmap for other systematically braided interventions to be delivered in child protective service settings.

## Methods

### Development of the SFH-SC braided curriculum: Steps 1 and 2 of systematic braiding

Program experts from both programs (*Smoke-Free Homes: Some Things are Better Outside* and *SafeCare*) completed the first two steps of the systematic braiding process prior to the pilot research. For step 1, experts identified the common content/pedagogical approaches of the two evidence-based programs, SafeCare and SFH. The selected programs were designed for similar populations and share theoretical underpinnings. Central to both programs is social cognitive theory [3, 64] and addressing behavioral change by targeting (1) behavioral capability (e.g., individual knowledge and skill change), (2) parental self-efficacy, (3) goal setting, and (4) environmental change. The programs differ with respect to mode of delivery (SafeCare home visiting vs SFH mailings and coaching call), dosage (SafeCare 18 weeks vs SFH 6 weeks), and targeted skills (see Table 1).

**Table 1:** Step 1: Systematic braiding. Shared elements of SafeCare and SFH

*Elements*	*SafeCare elements*	*Shared elements*	*Smoke-free home elements*
Participants	Parents of young children involved with child welfare	Parents of children 0–5 years who are at increased risk for SHS exposure	Targets low-income households with at least one smoker and at least one nonsmoker
Focus	Parent skill training for new parenting behaviors	Parent behavior change	Implement smoke-free home rules and change smoking behavior
Target for session delivery	Individual parent and child	Individual parent	Parent as household change agent
Mode of delivery	In-person at home	In-home	Telephone and mail to homes
Dosage	Weekly for 18–20 weeks	Brief	Once every 2 weeks for 6 weeks
Length	18 sessions	≥ 6 weeks	Four contacts in 6 weeks
Content	Explain, practice, model, and feedback approach to target behaviors for child safety and health and parent–child interactions	Structured curricula with protocol guidance for intervention deliverers. Parent-friendly content to help generalize skill change	“Five-Step Guide to a Smoke-Free Home” booklet, challenges and solutions booklet, stickers, signs pledges, newsletter, photonovella and coaching to set goals
Process assessment	Observational and satisfaction measures	Program evaluation embedded in delivery	Relevance, usefulness, satisfaction
Theoretical underpinnings	Social cognitive theory^97^	Social cognitive theory	Social cognitive theory, TTM^99^
Provider	Bachelor’s degree or above	Bachelor’s degree sufficient	None specified
Fidelity monitoring	Score audio recorded sessions with fidelity checklist	Audio recordings of coaching	Documentation of mailing sent, audio recording of coaching calls, and feedback

In step 2 of the systematic braiding process, the program expert team drafted a braided curriculum. Informed by step 1, we braided the full SFH curricula into the 6 sessions of both the Health and Safety modules of SafeCare. A family going through braided Smoke-Free Home and SafeCare (SFH-SC) curricula participated in either the Health or Safety module during their first 6 SafeCare sessions and receive the full dose of the SFH program. The family then continued receiving the remaining two modules of SafeCare in its standard form. During step 2 of the systematic braiding process, the program expert team discussed the importance of considering the potential negative impact of the new SFH content in homes where risk for violence is high and whether new content (e.g., requesting a smoker living in the home to engage in behavioral change) may place family members at additional risk for violence. As part of existing SafeCare Provider training, all SafeCare Providers are deeply trained in the management of family conflict and intimate partner violence prevention and intervention. Thus, while this certainly could be an important concern, SafeCare Providers know how to support these issues via assessment, problem-solving, and referral.

### SFH-SC pilot study: Step 3 of systematic braiding

Due to COVID-19 social distancing requirements and recent findings suggesting that the SafeCare Health module was the simplest to deliver virtually with fidelity [67], we piloted the SFH-SC version braided into the Health module with only maternal caregivers. No comparison group was used. See Table 2 for a description of the braided SFH-SC Health module.

**Table 2:** Step 2 of systematic braiding; outline of the braided SFH-SC curriculum

Week/session	SafeCare Health module Provider content and activities	Smoke-free home Provider content and materials
1	Formally assess parents’ baseline skills in one scenario from each type: emergency (ER), doctor’s appointment (DA), and care at home (CH). Discuss how to take a child’s temperature	Following SafeCare assessment, Provider to offer a brief overview of secondhand smoke and inform parent that they will receive useful resources during next session
2	Explain the Health Recording Chart and Sick and Injured Child Chart-Parent (SICC-P). Provider models the SICC-P steps with an ER scenario. Parent practices the SICC-P steps. Provider offers feedback. Repeat EMPF (explain, model, practice, feedback) with 2 new ER scenarios until parent achieves mastery	Provider to introduce STBO kit no. 1. Provider to deliver and review the materials in Kit no. 1 and ask parent to review them before the next session
3	Provider review the SICC-P and formally assess parents’ knowledge of ER scenarios from last session. Provider explains and models the SICC-P steps with a DA scenario. Parent practices the SICC-P steps with a DA scenario with Provider giving feedback. Repeat EMPF with 2 new DA scenarios until parent achieves mastery	Provider to review kit no. 1 with the parent if they did not review it on their own. Provider to ask parent how important it is to create a smoke-free home and work with parent to generate list of reasons to create a smoke-free home
4	Provider to review the SICC-P and formally assess parents’ knowledge of DA scenarios from last session. Explain and model the SICC-P steps with a CH scenario. Have the parent practice the SICC-P steps with a CH scenario with Provider giving feedback. Repeat EMPF with 2 new CH scenarios until parent achieves mastery	Provider to follow scripts from the confidence and stages of change checklist. Complete coaching protocol with goal setting related to relevant step in the five steps to a smoke-free home process, including training mothers as a change agent
5	Provider to review the SICC-P and formally assess parents’ knowledge of CH scenarios from last session. Repeat practice until mastery is achieved. Introduce prevention topic and utilize EMFP as needed for each prevention scenario. Repeat EMFP for Nutrition, Physical Activity, Immunization, and Preventing Abusive Head Trauma and Safe Sleep (infants) sections	Provider to ask about parent about progress toward achieving goal (e.g., family talk, setting a date). Provide to support parent in moving to next step (e.g., practice family talk, remove ashtrays, post signs, help set up safe smoking space outside). Provider to offer overview of challenges and solutions booklet and deliver kit no. 2; a photo novella that role models a family establishing a smoke-free home, and e-cigarette insert that encourages inclusion of vaping in the rule
6	Provider to formally assess parent on each scenario type (ER, DA, CH) and repeat EMFP until mastery achieved and provide feedback on parent progress since baseline	Provider to deliver kit no. 3 and provide a brief overview of the resources inside: newsletter, window cling, stickers, third hand smoke insert, etc

#### Caregiver Participants

Maternal caregivers were selected as the primary population for recruitment for the pilot study because they are most commonly the target participant of evidence-based home visitation programs like SafeCare. Caregivers were eligible for the study if they met the following inclusion criteria: (1) reported there was a smoker that lives in the home at least 3 or more nights a week (either herself or another family member), (2) were aged 18 years or older, and (3) were the primary caregiver of a child (aged 0 to 5 years) living in the home. Caregivers were excluded from the study if they reported that no smoker resided in the home.

Forty-nine maternal caregivers were referred from the partner sites (described in “Provider and procedures section”) and were contacted for screening to determine eligibility. After screening, we enrolled the first ten eligible participants. Recruitment ended after ten maternal caregivers consented and completed the baseline survey, and the study ended when the participants either dropped out or completed the intervention. We chose to end recruitment after 10 participants completed baseline measures based on practical considerations suggested in prior research, including budgetary constraints, the workflow for interventionists, and the number of participants needed to reasonably evaluate feasibility goals [70]. The average age for maternal caregiver participants was 32 (standard deviation [SD] = 11.8; Table 3), and the majority were non-Hispanic/Latinx (8 of 10) and Black or African American (8 of 10). Most mothers were single, never married (7 of 10), had completed a college degree (6 of 10), and were not currently employed (7 of 10). The number of children in the home ranged from 1 to 10. Two caregivers dropped out of the study, one immediately after the baseline survey and the second after 2 sessions of SFH-SC. Eight caregivers completed the baseline survey, the SFH-SC intervention, and the post-intervention survey.

**Table 3:** Systematic braiding steps 3 and 4: feasibility research phases, methods, and outcomes

Research phase	Methods and measures	Qualitative or quantitative	Feasibility area of focus^a^	Feasibility outcome^a^
Step 3: Systematic braiding: pilot braided curriculum with parents	Self-report survey	Quant	Limited efficacy	Pre-post changes in self-reported smoking, smoke-free home rules, and parent stress
	Semi-structured qualitative interview	Qual	Acceptability	Satisfaction, appropriateness, intent to continue use
			Demand	Interest and intent to use
			Practicality	Ability to carry out intervention activities
Step 4: Systematic braiding: gather feedback from Providers and modify curriculum	Semi-structured qualitative interview	Qual	Implementation	Factors that affect ease or difficulty of implementation, fit with SafeCare modules
			Practicality	Effects on participants, ability to carry out intervention activities
			Acceptability	Satisfaction, appropriateness, intent to continue use
			Demand	Perceived demand, interest and intent to use
	Brief Provider perceptions survey	Quant	Implementation	Implementation barriers, fit of SFH within SafeCare modules
			Acceptability	Appropriateness, intent to continue use
			Practicality	Effects on participants
			Demand	Intent to use (perception of parents), intent to attend trainings (for Providers)

Note: *Qual* qualitative data collected, *Quant* Quantitative data collected

^a^Feasibility areas of focus and outcomes are based on Bowen et al. (2009). How we design feasibility studies. *American Journal of Preventive Medicine*, *36*(5), 452–457

#### Caregiver Participant Procedures

All research was approved by the Institutional Review Board. Caregivers were recruited from three community partner sites including two medical clinics serving Medicaid populations and a government assistance program. Recruitment took place from May 7, 2021 through December 3, 2021. The research team used different recruitment methods based on COVID-19 protocols at each of the sites. At one clinic, we recruited in-person, and at another clinic, we posted flyers with study information on the back of exam room doors. We sent recruitment text messages to maternal caregivers involved in the government assistance program. Interested mothers either contacted or shared their contact information with the research team (either via Qualtrics or to the graduate research assistant doing in-person recruitment, depending on the partner site). Following consent, caregivers completed baseline measures via Zoom or telephone call, based on mothers’ preference. The baseline survey was completed in an interview style, in which the study staff asked the survey questions and entered the caregiver’s responses directly into REDCap. After completing the baseline survey, SFH-SC Providers contacted the mothers within 24 h to begin SFH-SC (6 weekly sessions). At the conclusion of SFH-SC, mothers completed a post-intervention survey and a brief qualitative interview, which took place between 6 and 8 weeks following the baseline survey. Mothers were compensated US $35 for the baseline survey, US $10 for each completed SFH-SC session, and US $40 for the post-intervention survey and interview, a total of up to US $135.

The SFH-SC Providers for the pilot were two Senior Training Specialists at the National SafeCare Training and Research Center, each with more than 8 years of SafeCare experience. A SFH program expert trained Providers on SFH intervention and monitored intervention delivery for fidelity. Specifically, the SFH trainer listened to recordings of the braided SFH-SC sessions and provided feedback and support to ensure that the SFH portion was implemented as planned. SFH-SC included six virtually delivered training sessions (Table 2).

#### Caregiver Participant Measures

##### Smoke-free home rules

Items assessing smoking in the home included the following: 1) “Which statement best describes the rules about smoking inside your home (response options included: “smoking is…: is not allowed anywhere; is allowed in some places or at some times; smoking is allowed anywhere; or there are no rules?” 2) “What smoking products are covered by the rules? (response options included: smoke from cigarettes, cigars/cigarillos, marijuana, vapor/aerosol from Electronic Nicotine Delivery Systems [ENDs])” 3) “How often are your smoking rules broken by someone? (never, rarely, sometimes or very often)” [42, 43]

##### Smoking habits

We asked three items to assess smoking habits of the participant and others living in the home. Items included the following: “Are you a current smoker?” (*Yes, no*); “Do you live in the same house as someone who smokes?” (*Yes, no; note: we did not ask who the smoker was if the response to this item was yes*); and the number of smokers in the home.

##### Caregiver stress

Parenting stress was measured by the validated Parent Stress Index-Short Form (PSI-SF), a 36-item self-report measure [1, 33]. The PSI-SF consists of three subscales (i.e., parental distress, parent–child dysfunctional interactions, and difficult child) made up of 12 items each, as well as a subscale to measure a parent’s defensive responding (7 items). For each item, parents indicate the degree to which they agree or disagree with the statement by choosing one of the five response options on a Likert scale, with higher numbers indicating more parent stress.

##### Demographics

Mothers completed information documenting their age, gender, race, ethnicity, relationship status, education level, employment, income, and number of children in the home.

#### Caregiver SFH-SC intervention experience and satisfaction

Maternal participants completed a 1-h qualitative interview with study staff about their thoughts and experiences with SFH-SC. Study staff asked maternal participant questions specific to their attitudes toward SFH-SC, such as “What was it that you liked or disliked about the parenting program in general?” or “What was it that you liked or disliked about the added smoke-free home material?” Maternal participants were also asked how they felt discussing smoking habits and smoke-free home rules with SFH-SC specialists. This included questions such as “What helped to make it safe to talk about these [smoking] habits?” and “Did including a focus on creating a smoke-free home impact your engagement in any way?” Lastly, participants were asked to provide some suggestions for how future SFH-SC Providers could best navigate working with families who do have smokers in the home. Interviews were audio recorded.

### Provider review of SFH-SC curriculum: Step 4 of systematic braiding

The purpose of the curriculum review is to get Provider feedback on the novel SFH-SC curriculum.

#### Provider Participants

Providers were eligible to participate in this phase of the project if they were certified in SafeCare and were working in a US child protection or prevention service settings within state that has CDC-documented high rates of tobacco use. Recruitment took place from December 2021 through February 2022 and was stopped when 9 Providers consented to the study. Providers interviewed had an average of 6 years of SafeCare experience (range = 2 to 14 years).

#### Provider Procedures

An email was sent via the National SafeCare Training and Research Center Provider listserv. The recruitment email offered information on the inclusion criteria and the curriculum review study. Providers were invited to respond to the email if they were interested. A member of the research team contacted the first 10 Providers who responded to the initial recruitment email and consented the Providers via Zoom. Providers received an electronic version of the SFH-SC curriculum (including the SFH materials that go to families) via email and were also mailed a hard copy and were asked to review the materials over a 2-week period. After the review period, the 9 US SafeCare-certified Providers completed the 2-h review of the SFH-SC braided curriculum and participated in a subsequent 1-h feedback interview with study staff. Providers were compensated with a US $200 Amazon gift card.

#### Provider Measures

##### SFH-SC curriculum feedback interview

Study staff asked Providers about their overall impression of the braided curriculum, their perception of the utility of a smoke-free home intervention for their clients, and the benefits of a smoke-free home intervention. Providers were also asked to share their thoughts on how well the SFH materials fit into each SafeCare module (safety, health, parent–child interaction). Finally, Providers shared feedback about their perceived potential barriers and facilitators to delivering SFH-SC. Surveys were audio recorded.

##### SFH-SC feasibility

Providers completed a brief survey through REDCap that assessed the feasibility of SFH-SC after they completed the curriculum review and follow-up interview. The survey consisted of 4 items that assessed feasibility of SFH-SC, including Provider perceptions of (1) how receptive families with a smoker in the home would be to SFH-SC, (2) the potential impact of SFH-SC on creating a smoke-free home, (3) Provider openness to participate in additional training for SFH-SC, and (4) how well the SFH materials fit with each of the modules of SafeCare. Response options were on a 5-point Likert scale ranging from 1 (very difficult) to 5 (very easy). The overall score ranged from 4 to 20; higher scores indicate greater feasibility.

### Data analysis

Descriptive statistics were computed using SAS 9.4 (SAS Institute Inc., 2013) for the quantitative data collected in steps 3 and 4, which included the maternal demographics, smoke-free home rules, smoking habits, parent stress, and Provider feasibility survey. Quantitative analyses included only those who completed the intervention (*n* = 8) to understand if the measures selected were sensitive enough to detect changes in smoke-free home rules and behaviors and parenting stress following the intervention (SFH-SC); however, no statistical tests were conducted, as the aim of the pilot was to assess the feasibility of SFH-SC [22]. The qualitative interviews collected with caregivers in step 3 were transcribed by members of the research team, and a thematic analysis was conducted to summarize key themes. The qualitative data collected with participants in step 4 was summarized into key points for each question and reviewed by two study staff.

## Results

### SFH-SC pilot study results with maternal caregivers: Step 3 of systematic braiding

#### Caregiver Demographics

The demographics of caregivers who participated in the pilot are summarized in (Table 4).

**Table 4:** Demographic characteristics of caregivers participating in the SFH-SC pilot (*N* = 10)

Variable	*n* (%)
Age, mean (SD)	32.2 (11.8)
Race	
African American/Black	8 (80.0)
Asian/Asian American	0 (0.0)
Caucasian/White	1 (10.0)
Pacific Islander	1 (10.0)
Ethnicity	
Hispanic/Latinx	2 (20.0)
Non-Hispanic/Latinx	8 (80.0)
Current employment	
Yes	3 (30.0)
No	7 (70.0)
Monthly income	
US $0–249	0 (0.0)
US $250–499	1 (10.0)
US $500–999	3 (30.0)
US $1000–1999	2 (20.0)
US $2000 or more	2 (20.0)
Prefer not to answer	2 (20.0)
Number of children	
1	7 (70.0)
3	2 (20.0)
10	1 (10.0)
Age of children, mean (SD)^a^	5.6 (6.0)

^a^Observed range, 4 days to 17 years

#### Smoking habits and smoke-free home rules

In the baseline survey, 2 of the 8 caregiver participant completers reported being current smokers (Table 5). Seven of the 8 participants lived in the same household with someone else who smoked. In terms of existing smoke-free home rules, half of the participants reported having a full smoking ban in the home, and the other half reported that smoking was allowed in some places or at some times in the home. There were small increase in caregiver-reported smoke-free home rules from baseline to the 8-week post-intervention survey among study completers.

#### Parent stress

Average reductions from baseline to post-intervention ranged from 1.0 (*SD* = 7.3) to 2.5 (*SD* = 4.6) across subscales (Table 5). The average reduction from baseline to post-intervention for the PSI-SF total score was 5.9 (*SD* = 10.2).

**Table 5:** Self-reported feasibility outcomes for limited efficacy among caregivers who completed the SFH-SC pilot

Variable	Baseline (*n* = 8)	Post-SFH SC (*n* = 8)	Mean difference
Smoking questions [no. of participants (%)]			
Current smoker			
Yes	2 (25.0)	2 (25.0)	
No	6 (75.0)	6 (75.5)	
Live in the same household with someone who smokes			
Yes	7 (87.5)	8 (100.0)	
No	1 (12.5)	0 (0.0)	
Number of smokers in the home			
1	7 (87.5)		
2	1 (12.5)		
Smoke-free home rules			
Current enforcement of smoke-free home rules			
Not allowed anywhere inside	4 (50.0)	5 (62.5)	
Allowed in some places or at some times	4 (50.0)	3 (37.5)	
Allowed anywhere inside	0 (0.0)	0 (0.0)	
No smoke-free home rules	0 (0.0)	0 (0.0)	
Products covered by smoke-free home rules*			
Smoke from cigarettes	7 (87.5)	8 (100)	
Cigars/cigarillos	4 (50.0)	5 (62.5)	
Marijuana	4 (50.0)	6 (75.0)	
Vapor/aerosol from ENDS	5 (62.5)	6 (75.0)	
Frequency of smoking rules being broken			
Never	3 (37.5)	3 (37.5)	
Rarely	3 (37.5)	3 (37.5)	
Sometimes	2 (25.0)	0 (0.0)	
Very often	0 (0.0)	2 (25.0)	
Parenting stress inventory [mean (SD)]			
Defensive responding subscale^a^	15.6 (4.7)	14.3 (5.6)	1.4 (2.1)
Parental distress^b^	45.8 (6.7)	22.4 (8.1)	2.4 (4.7)
Parent–child dysfunctional interaction^c^	18.5 (4.9)	16.0 (5.3)	2.5 (4.6)
Difficult child^d^	26.3 (11.4)	25.3 (9.3)	1.0 (7.3)
Total score^e^	69.5 (21.3)	63.6 (18.8)	5.9 (10.2)

*ENDS* electronic nicotine delivery system. ^a^Summary of 7 items; possible range, 7–35. ^b^Possible range, 12–60. ^c^Possible range, 12–60. ^d^Possible range, 12–60. ^e^Possible range: 136–180

###  Caregiver SFH-SC intervention experience and satisfaction

#### SafeCare and smoke-free home materials

All participants indicated they thought the SafeCare health materials and health manual are beneficial for parents. They also noted that the SFH materials were very informative, particularly regarding how smoking affects children’s health. One participant stated the following:

I definitely think it’s beneficial and helpful to people who don’t understand the long-term effects of smoking in the home just like myself. For instance, I felt as long as we don’t smoke around the kids…then it shouldn’t be a concern and that’s not true.

Some participants (*n* = 2) who had taken steps to create a smoke-free home prior to the study felt that the smoke-free home materials confirmed that taking those steps were correct and that those steps should be acknowledged or celebrated. Participants mentioned that the smoke-free home component and materials helped to create, enforce, and reinforce smoke-free home rules (*n* = 3) and helped to begin conversations about smoking and provide approachable materials with helpful information to the smoker in the home (*n* = 5). For example, one participant noted as follows:

In the past, I would say [to the other smoker in the home] smoke in the other room, but now I see that even if you’re in the other room or in the house in general it can still affect people and to me that was [a] change in my thinking for how serious it was, so that’s really what pushed me to do it [establish smoke-free home rules].

Another participant indicated the following:

It was really helpful for me to have those materials cause it was kind of like a way for start to have those conversations with my dad… it was helpful for like a conversation that would’ve been hard for me to have otherwise.

#### Discussing smoke-free home rules with the SafeCare Provider

Overall, despite the sensitive nature of the topic, most participants reported having positive experiences when discussing the impact of having a smoke-free home on child health and establishing smoke-free rules with the SafeCare Provider (*n* = 7). In fact, two of the participants mentioned that the most helpful part of the SFH-SC program was discussing smoking in the home and options to create a smoke-free home with the Provider. When participants were asked about their experiences talking to the SafeCare Provider about smoking in the home, they reported that they felt comfortable because the Providers were easy to talk to, open-minded, and non-judgmental. One participant described as follows:

It felt like a judgement-free zone. There was no judgement. I don’t feel like…[the providers responses] made me feel like I could talk to [them] about the smoking habits in general.

Participants emphasized the critical importance of Providers being non-judgmental and speaking in a neutral tone to avoid shaming smoking behaviors. Participants suggested that for future SFH-SC delivery, Providers should avoid pressuring parents to make certain changes or coming across as condescending or seeming like an expert. One participant offered a summary of these recommendations, stating the following:

It’s all about your approach, the tone you use, so if you have sort of a neutral approach, neutral tone and it doesn’t come off as badgering or act like you know it all or you’re better than them because you don’t do certain things then they’ll receive it in a good manner.

#### Participant engagement in Smoke-Free Home SafeCare

Participant completers reported the content was highly engaging. One participant stated the following:

I really was like crunched for time… but I always found time even if I had to reschedule a few times to have those meetings with [the provider] because I really enjoyed the program and I knew I was being informed with things that I may not have known….

Participants provided recommendations to enhance the program, including involving all caregiver parties (i.e., smoker in the home if the smoker is not the caregiver partaking in the program), allowing children to be a part of or have a say in the program, offer education on other coping mechanisms to relieve stress, and provide resources for individuals with financial issues or other struggles.

### Provider review of SFH-SC curriculum: Step 4 systematic braiding

#### General feedback on the braided curriculum

During the interview after reviewing the braided curriculum, Providers indicated that they were interested in SFH-SC, even if SHS exposure was not a particular concern for the majority of families they serve, because they felt the added SFH content complemented the goals of SafeCare. They also appreciated having this topic integrated into SafeCare for families where there is a smoker in the home, with one participant stating the following:

Having [materials about SHS prevention already] integrated so we don’t have to pull resources ourselves would be helpful. A combined intervention is a double win.

When considering the SFH content fit with the SafeCare modules, Providers indicated that they thought the SFH materials did not align well with the parent–child interaction module but fit very well with the Health and Safety modules. The Providers made suggestions for ways to further integrate the SFH content into the SafeCare sessions, by spending some time going over all materials that are provided to parents on the topic versus asking parents to review on their own. The Providers indicated that taking the time to discuss SFH content with parents would complement the active delivery structure of SafeCare in which content is explained and modeled by the Provider followed by parents’ practice with corrective feedback as needed. Following the same active delivery structure was suggested to help make SFH-SC feel like one integrated program to the parent.

#### Program engagement

Providers noted the sensitive nature of discussing smoking and smoking in the home with caregivers and were concerned about how this discussion could impact family engagement in SafeCare. Participants mentioned that it would be critical to build a strong rapport with the family before launching into the complex topics addressed in SFH-SC. One Provider noted the following:

[In terms of] engagement, it would come down to how you do it. It is more of a how you do it than a what it is… Conversational prompts about how to introduce these materials would be helpful.

#### SafeCare Provider SFH-SC feasibility survey

All SafeCare Providers reported that the Smoke-Free Home SafeCare would improve clients’ abilities to create a smoke-free home (*M* = 4.55; *SD* = 0.53). Additionally, most SafeCare Providers (78%) reported that they would be willing to participate in additional training to be certified in SFH-SC (*M* = 4.11; *SD* = 1.05). The majority of Provider participants indicated that the SFH materials fit best in the Safety module (89%) or the Health module (78%) and least with the parent–child interaction module (*M* = 2.22; *SD* = 1.48). Over half of the Providers (56%) indicated that the curriculum would be easy to deliver as designed. Overall, SafeCare Providers indicated that they thought the SFH-SC curriculum was feasible (*M* = 4.0 [max = 5]; *SD* = 0.59).

## Discussion

The study purpose was to fill a gap in existing intervention science by using an innovative approach (systematic braiding) to leverage existing evidence-based interventions to address two significant public health issues among young children. The first four steps of systematic braiding were successfully completed. Results from the SFH-SC pilot study suggest that maternal caregivers benefited from participation in SFH-SC and were engaged and satisfied with the program. Results from the Providers’ SFH-SC curriculum review suggest that a few additional curriculum modifications were needed to finalize SFH-SC for further evaluation (i.e., type 1 hybrid trial). Furthermore, Providers consistently reported that the program would be feasible and includes very important content for families at risk, including those involved in child protection services.

### SFH-SC Caregiver Outcomes

In the pilot, 80% of caregivers were retained through SFH-SC intervention delivery and completed both baseline and post-intervention surveys. These results are promising, given that retaining parents in prevention program research often proves challenging. For example, prior SafeCare studies reported retention rates of participants ranging from 50% [17] to 61% [66], respectively, though the timing of these studies is generally much longer (up to 1 year) than in this pilot. The retention rates are also important to consider within the context and timing of the trial, which took place in the midst of the COVID-19 pandemic in 2021–2022. Maternal caregiver participants were socioeconomically disadvantaged, recruited from community partners that serve families living in under-resourced communities. The success in retaining the population is promising for future studies examining the effectiveness of the braided curriculum.

Most mothers recruited for the trial were not smokers themselves (4 out of 10 recruited reported smoking, 2 of the 8 completers) but were living in the home with someone else who smoked (9 out of the 10 recruited, all 8 completers). Notably, of the two participants who identified as a smoker at baseline, one dropped out of the study. For the primary outcome, smoke-free home rules, no substantial changes emerged from the baseline to post-intervention surveys. This was surprising, given prior effectiveness observed in the SFH intervention [39, 40]. One explanation for this could be that the participant themselves was not necessarily the smoker in the home. Family dynamics and the sensitive nature of smoking could pose barriers to establishing smoke-free home rules. For example, if a maternal participant lives with an elder family member who smokes and pays for the home, it may be more challenging to establish smoke-free home rules than it would be for a participant who is the smoker and pays for housing themselves. Furthermore, upon closer examination of the pilot study results, a critical limitation in the study inclusion criteria was illuminated. Specifically, 50% of the sample reported that they already had a full smoking ban in their home, and all participants reported at least some smoke-free home rules were already in place. Based on this feasibility work, we will add an additional inclusion criteria in our future studies such that only participants who allow smoking in the home will be eligible to participate.

A meta-analysis by Kaminski et al. [80] found that as parenting programs get more diffuse, the impact on target outcomes can be negatively impacted. We explored parenting outcomes, but as the purpose was to demonstrate acceptability and feasibility, we did not examine efficacy. Mothers reported reductions in parenting stress from the baseline to post-intervention, consistent with finding in prior SafeCare published studies (e.g., [77]). Our finding of reduced stress is promising and suggests that the braided program did not negatively impact the primary parenting outcomes targeted by SafeCare. Furthermore, this suggests the SafeCare active ingredients were maintained even with the integration of the smoke-free home materials in this preliminary work.

Caregiver participant completers’ feedback in the qualitative interviews suggested they thought the materials for both SafeCare and SFH were useful for parents. While mothers acknowledged that having conversations about smoking in the home can be a sensitive topic, they noted that the smoke-free home materials reinforced the rules they had implemented and offered effective approaches to sharing this information with other smokers living in the home. Mothers indicated that their conversations with SafeCare Providers about creating a smoke-free home were very useful and proceeded in a non-judgmental way. This is particularly important as prior research with nationally implemented home visiting prevention programs found that Providers did not feel well-prepared to address parental tobacco use, with concerns it would negatively affect program engagement [20]. The braided SFH-SC curriculum was well-accepted and did not appear to negatively impact parent program engagement for the completers in our pilot, as noted by the high rates of completion and mothers’ qualitative responses. Collectively, these data suggest high feasibility and relevance for the braided intervention.

### SafeCare Providers SFH-SC Feedback

In terms of SafeCare Provider curriculum feedback, participants noted that SFH compliments the goals of SafeCare, with both targeting the creation of a safe and stable home environment for young children. This is commensurate with an ongoing movement in the child maltreatment field that underscores the utility of prevention programs that could address diverse risks simultaneously to promote child well-being and safety [80]. Providers commented that having the smoke-free materials integrated into SafeCare could save them time by reducing the need for them to search for additional resources to prevent SHS with the families they serve.

Providers reported that they would be willing to participate in workshop training and implement the program with families for SFH-SC if this program was to become readily available. They stated both in interviews and on the feasibility survey that the SFH content fits best in the SafeCare Health and Safety modules. Providers also noted the importance of building a strong rapport with mothers before tackling topics related to smoking and tobacco use, which was commensurate with feedback offered by mothers in the pilot. Accordingly, training on rapport building and non-judgmental approaches to encourage parent engagement in SFH-SC will be highlighted in the Provider workshop training for the hybrid trial.

Providers did have some suggestions for curriculum modifications regarding how SFH could be further integrated into SafeCare. Specifically, they recommended the research team consider deeper discussion of the SFH materials as part of the braided session, as well as utilizing the standard SafeCare approaches (e.g., Provider modeling and parent practice) when reviewing the materials with parents. These were important considerations; however, a decision was ultimately made to maintain the fidelity of the original SFH materials in the final braided curriculum for several reasons. First, in its evidence-based, original delivery format (mailings and one coaching phone call), SFH has made a strong impact on smoke-free home rules in randomized trials [39, 40]. Second, the child welfare workforce is already experiencing high job-related demands and is often under resourced [65]. Since the future trial is planned to be delivered in two states where SafeCare is implemented in child protection services, we attempted to avoid as much workload burden for Providers as possible while maintaining the integrity of the two original programs. Including some of the Provider recommendations, such as additional psychoeducation and modeling of some of the smoke-free home content, could greatly extend session duration. Lastly, the potential scalability and public health impact of SFH-SC, if found to be effective, are a major goal for this research,thus, the grant funding does not support substantial changes to the time for delivery of SFH braided into SafeCare as part of child protective system implementations. Future research could expand on this work to explore adaptations to further optimize the delivery of SFH-SC [32].

### Limitations

The formative work described herein is promising and will be useful to future teams working on systematic braiding efforts, but is not without limitation. First, there was a small number of participants who participated in this pilot work. Accordingly, there was no power to assess statistically significant differences in outcomes or explore potential moderators/mediators related to outcome change, and the generalizability of the findings is limited. Second, while the caregiver participants were experiencing some risk factors related to socioeconomic status, none was involved in child protections services, an inclusion criterion in the upcoming hybrid trial, which may impact program engagement and retention and further limits the generalizability of the current findings. Third, there were no control participants included to allow for comparison on the outcome variables. Fourth, the reliance of self-report measures for both the smoke-free home and parenting outcomes are subject to social desirability bias. To address some of these sources of bias in subsequent research, we will use air monitors to corroborate the self-report of smoke-free home rules. Fifth, the current assessment measures did not identify who the tobacco user was in the home if it was not the primary caregiver. This is important to ask in future research because who the tobacco user is may have significant implications for success in establishing smoke-free home rules.

## Conclusions, next steps, and future directions

Upwards of 3.5 million children in the USA are the subjects of a child protection report each year [72]. Parents of these children access services, such as SafeCare, while they are involved with the child protection system. Evidence-based interventions designed to simultaneously target multiple risk factors that negatively affect child health and well-being, such as child maltreatment and SHS, could have a meaningful public health impact for at-risk families. Furthermore, using innovative approaches to leverage complementary evidence-based interventions that tackle different public health outcomes, is imperative to advance the field of intervention science, maximizing the outcomes of efforts that are costly to deliver and are burdensome in terms of time. Systematic braiding is one promising, innovative approach that could be used to advance the field of intervention science and to create cost-effective public health impact through the improved, efficient dissemination of evidence-based interventions to create clinically meaningful changes for children and families and improve health equity.

Future directions in this work include a Hybrid type 1 trial, funded by the National Cancer Institute, where SafeCare Providers working in child protective and prevention service systems in the USA will be recruited and randomly assigned to either SFH-SC (offered in either the SafeCare Health or Safety module) or Standard SafeCare. Providers will each serve 10 research families (*N* = 500) who report a smoker living in the home and no current smoke-free home rules. If the sample allows, we will explore variables, including who the tobacco user is in the home, as potential moderators of program engagement, dropout, and the smoke-free home rule outcomes. The primary outcome, smoke-free home status, will be measured via self-report, and smoke-free home rules will be validated via air nicotine monitors. Process measures will be collected to examine how SFH-SC impacts Provider fidelity, delivery time and costs, and parent engagement.

## Supplementary Information

**Additional file 1.** Smoke Free Home SafeCare – Pilot and Feasibility Study.

**Additional file 2.** CONSORT checklist of information to include when reporting a pilot trial*.

## Data Availability

The datasets used and/or analyzed during the current study are available from the corresponding author on reasonable request.

## Abbreviations

SHS: Secondhand tobacco smoke
SFH-SC: Smoke-Free Home SafeCare
SES: Socioeconomic status

## References

[1] Abidin RR (1995). Parenting stress index, Third Edition: Professional Manual.

[2] Anderson TM, Lavista Ferres JM, Ren SY, Moon RY, Goldstein RD, Ramirez JM, & Mitchell EA. Maternal smoking before and during pregnancy and the risk of sudden unexpected infant death. Pediatrics. 2019;143(4):e20183325. p 1–8. 10.1542/peds.2018-3325 PMC6564075 30858347

[3] Bandura A (1989). Human agency in social cognitive theory. American Psychiatry..

[4] Barboza GE (2019). The geography of child maltreatment: a spatiotemporal analysis using Bayesian hierarchical analysis with integrated nested Laplace approximation. J Interpers Violence.

[5] Behrman JR, Calderon MC, Preston SH, Hoddinott J, Martorell R, Stein AD (2009). Nutritional supplementation in girls influences the growth of their children: prospective study in Guatemala. Am J Clin Nutr.

[6] Biener L, Cullen D, Di ZX, Hammond SK (1997). Household smoking restrictions and adolescents’ exposure to environmental tobacco smoke. Prev Med.

[7] Brown DW, Anda RF, Felitti VJ, Edwards VJ, Malarcher AM, Croft JB, Giles WH (2010). Adverse childhood experiences are associated with the risk of lung cancer: a prospective cohort study. BMC Public Health.

[8] Bruce J, Gunnar MR, Pears KC, Fisher PA (2013). Early adverse care, stress neurobiology, and prevention science: lessons learned. Prev Sci.

[9] Bundy ŁT, Haardörfer R, Kegler MC, Owolabi S, Berg CJ, Escoffery C, … & Kreuter MW. Disseminating a smoke-free homes program to low socioeconomic status households in the United States through 2-1-1: results of a national impact evaluation.Nicotine Tob Res. 2018;22(4):498-505. 10.1093/ntr/nty256 PMC7368345 30517679

[10] Campbell F, Conti G, Heckman JJ, Moon SH, Pinto R, Pungello E, Pan Y (2014). Early childhood investments substantially boost adult health. Science.

[11] Carta JJ, Lefever JB, Bigelow K, Borkowski J, Warren SF (2013). Randomized trial of a cellular phone-enhanced home visitation parenting intervention. Pediatrics.

[12] Chaffin M, Bard D, Bigfoot DS, Maher EJ (2012). Is a structured, manualized, evidence-based treatment protocol culturally competent and equivalently effective among American Indian parents in child welfare?. Child Maltreat.

[13] Chaffin M, Hecht D, Bard D, Silovsky JF, Beasley WH (2012). A statewide trial of the SafeCare home-based services model with parents in child protective services. Pediatrics.

[14] Collaco JM, Aherrera AD, Ryan T, McGrath-Morrow SA (2014). Secondhand smoke exposure in preterm infants with bronchopulmonary dysplasia. Pediatr Pulmonol.

[15] Cook CR, Frye M, Slemrod T, Lyon AR, Renshaw TL, Zhang Y (2015). An integrated approach to universal prevention: independent and combined effects of PBIS and SEL on youths’ mental health. Sch Psychol Q.

[16] Corso PS, Edwards VJ, Fang X, Mercy JA (2008). Health-related quality of life among adults who experienced maltreatment during childhood. Am J Public Health.

[17] Damashek A, Doughty D, Ware L, Silovsky J (2011). Predictors of client engagement and attrition in home-based child maltreatment prevention services. Child Maltreat.

[18] Dong M, Giles WH, Felitti VJ, Dube SR, Williams JE, Chapman DP, Anda RF (2004). Insights into causal pathways for ischemic heart disease: adverse childhood experiences study. Circulation.

[19] Doyle O, Harmon CP, Heckman JJ, Tremblay RE (2009). Investing in early human development: timing and economic efficiency. Econ Hum Biol.

[20] Duggan A, Portilla XA, Filene JH, Crowne SS, Hill CJ, Lee H & Knox V. Implementation of evidence-based early childhood home visiting: results from the mother and infant home visiting program evaluation. OPRE Report 2018–76A. 2018. https://www.acf.hhs.gov/sites/default/files/opre/mihope_implementation_execsummary_2018_10_26_508.pdf.

[21] Dube SR, Fairweather D, Pearson WS, Felitti VJ, Anda RF, Croft JB (2009). Cumulative childhood stress and autoimmune diseases in adults. Psychosom Med.

[22] Eldridge SM, Chan CL, Campbell MJ, Bond CM, Hopewell S, Thabane L, & Lancaster GA. CONSORT 2010 statement: extension to randomised pilot and feasibility trials. BMJ. 2016;355:i5239. 10.1136/bmj.i5239 PMC5076380 27777223

[23] Felitti V, Anda RF, Nordenberg D, et al. Relationship of childhood abuse and household dysfunction to many of the leading causes of death in adults: The Adverse Childhood Experiences (ACE) Study. Am J Prev Med. 1998;14(4):245–58. 10.1016/S0749-3797(98)00017-8. 10.1016/s0749-3797(98)00017-8 9635069

[24] Felitti VJ, Anda RF, Nordenberg D, Williamson DF, Spitz AM, Edwards V, … & Marks JS. Reprint of: relationship of childhood abuse and household dysfunction to many of the leading causes of death in adults: the adverse childhood experiences (ACE) study. Am J Prev Med. 2019;56(6):774–786. 10.1016/j.amepre.2019.04.001 31104722

[25] Fergusson DM, Boden JM, Horwood LJ (2008). Exposure to childhood sexual and physical abuse and adjustment in early adulthood. Child Abuse Negl.

[26] Gartner CE, Hall WD (2013). Is the socioeconomic gap in childhood exposure to secondhand smoke widening or narrowing?. Tob Control.

[27] Gehrman CA, Hovell MF (2003). Protecting children from environmental tobacco smoke (ETS) exposure: a critical review. Nicotine Tob Res.

[28] Gertler P, Heckman J, Pinto R, Zanolini A, Vermeersch C, Walker S, Chang SM, Grantham-McGregor S (2014). Labor market returns to an early childhood stimulation intervention in Jamaica. Science (New York, N.Y)..

[29] Gilbert R, Widom CS, Browne K, Fergusson D, Webb E, Janson S (2009). Burden and consequences of child maltreatment in high-income countries. Lancet (London, England).

[30] Guastaferro K, Miller K, Lutzker JR, Whitaker DJ, Chatham JS, Lai BS, Kemner,  (2017). Implementing a braided home-based parent-support curriculum: lessons learned. Psychosoc Interv.

[31] Guastaferro K, Zadzora KM, Reader JM, Shanley J, Noll JG (2019). A parent-focused child sexual abuse prevention program: development, acceptability, and feasibility. J Child Fam Stud.

[32] Guastaferro K, Strayhorn JC, Collins LM (2021). The multiphase optimization strategy (MOST) in child maltreatment prevention research. J Child Fam Stud.

[33] Haskett ME, Ahern LS, Ward CS, Allaire JC (2006). Factor structure and validity of the parenting stress index-short form. J Clin Child Adolesc Psychol.

[34] Hoddinott J, Alderman H, Behrman JR, Haddad L, Horton S (2013). The economic rationale for investing in stunting reduction. Matern Child Nutr.

[35] Hoddinott J, Maluccio JA, Behrman JR, Flores R, Martorell R (2008). Effect of a nutrition intervention during early childhood on economic productivity in Guatemalan adults. Lancet (London, England).

[36] Holtby S, Zahnd E, Grant D, & Park R. Children’s exposure to secondhand smoke: nearly one million affected in California. Policy brief (UCLA Center for Health Policy Research), (PB2011–9), 2011:1–8. 22097395

[37] Homa DM, Neff LJ, King BA, Caraballo RS, Bunnell RE, Babb SD, … & Wang L. Vital signs: disparities in nonsmokers’ exposure to secondhand smoke—United States, 1999–2012. Morbid Mortal Wkly Rep. 2015;64(4):103. PMC4584848 25654612

[38] Horwitz AV, Widom CS, McLaughlin J, & White HR. The impact of childhood abuse and neglect on adult mental health: a prospective study. J Health Soc Behav. 2001;42(2):184–201. 11467252

[39] Kegler MC, Bundy L, Haardörfer R, Escoffery C, Berg C, Yembra D, Kreuter M, Hovell M, Williams R, Mullen PD, Ribisl K, Burnham D (2015). A minimal intervention to promote smoke-free homes among 2-1-1 callers: a randomized controlled trial. Am J Public Health.

[40] Kegler MC, Haardörfer R, Bundy LT, Escoffery C, Williams RS, Hovell M, Kreuter M, Mullen PD (2019). Moderators of establishing a smoke-free home: pooled data from three randomized controlled trials of a brief intervention. J Community Health.

[41] Kelly A, Denning-Kemp G, Geiringer K, Abdulhamid A, Albabtain A, Beard M, … & Baker MG. Exposure to harmful housing conditions is common in children admitted to Wellington Hospital. NZ Med J (Online). 2013;126(1387):108–26. 24362739

[42] King BA, Patel R, Babb SD, Harman AM, Freeman A (2016). National and state prevalence of smoke-free rules in homes with and without children and smokers: two decades of progress. Prev Med.

[43] Kruger J, Jama A, Homa DM, Babb SD, King BA (2015). Smoke-free home and vehicle rules by tobacco use status among U.S. adults. Prev Med.

[44] Lanier P, Jonson-Reid M, Stahlschmidt MJ, Drake B, Constantino J (2010). Child maltreatment and pediatric health outcomes: a longitudinal study of low-income children. J Pediatr Psychol.

[45] Lecomte T, Corbière M, Simard S, Leclerc C (2014). Merging evidence-based psychosocial interventions in schizophrenia. Behav Sci.

[46] Lefever JEB, Bigelow KM, Carta JJ, Borkowski JG, Grandfield E, McCune L, … & Warren SF. Long-term impact of a cell phone–enhanced parenting intervention. Child Maltreat. 2017;22(4):305–314. 10.1177/1077559517723125 28845676

[47] Liu Y, Merritt DH (2018). Familial financial stress and child internalizing behaviors: the roles of caregivers’ maltreating behaviors and social services. Child Abuse Negl.

[48] Longman JM, Passey ME (2013). Children, smoking households and exposure to second-hand smoke in the home in rural Australia: analysis of a national cross-sectional survey. BMJ Open.

[49] National Cancer Institute Evidence-Based Cancer Control Programs (NCI EBCCP). Smoke free homes: some things are better outside. U.S. Department of Health and Human Services. 2020.

[50] Maguire-Jack K (2014). Multilevel investigation into the community context of child maltreatment. J Aggress Maltreat Trauma.

[51] Maluccio JA, Hoddinott J, Behrman JR, Martorell R, Quisumbing AR, Stein AD (2009). The impact of improving nutrition during early childhood on education among Guatemalan adults. Econ J.

[52] McLeigh JD, McDonell JR, Lavenda O (2018). Neighborhood poverty and child abuse and neglect: the mediating role of social cohesion. Child Youth Serv Rev.

[53] Merianos AL, Jandarov RA, Choi K, Mahabee-Gittens EM (2019). Tobacco smoke exposure disparities persist in US children: NHANES 1999–2014. Prev Med.

[54] Metayer C, Zhang L, Wiemels JL, Bartley K, Schiffman J, Ma X, … & Buffler PA. Tobacco smoke exposure and the risk of childhood acute lymphoblastic and myeloid leukemias by cytogenetic subtypetobacco smoking and the risk of childhood leukemia subtypes. Cancer Epidemiol Biomark Prev. 2013;22(9):1600–1611. 10.1158/1055-9965.EPI-13-0350 PMC3769478 23853208

[55] Molnar BE, Goerge RM, Gilsanz P, Hill A, Subramanian SV, Holton JK, … & Beardslee WR. Neighborhood-level social processes and substantiated cases of child maltreatment. Child Abuse Neglect. 2016;51:41–53. 10.1016/j.chiabu.2015.11.007 PMC4713333 26684963

[56] Mullen PD, Savas LS, Bundy ŁT, Haardörfer R, Hovell M, Fernández ME, Monroy JA, Williams RS, Kreuter MW, Jobe D, Kegler MC (2016). Minimal intervention delivered by 2-1-1 information and referral specialists promotes smoke-free homes among 2-1-1 callers: a Texas generalization trial. Tob Control.

[57] National Center for Chronic Disease Prevention and Health Promotion (US) Office on Smoking and Health. The health consequences of smoking—50 years of progress: a report of the surgeon general. Centers for Disease Control and Prevention (US). 2014. 24455788

[58] National Research Council (US) and Institute of Medicine (US) Committee on Integrating the Science of Early Childhood Development. Shonkoff JP, & Phillips DA. Eds. From Neurons to Neighborhoods: The Science of Early Childhood Development. National Academies Press (US). 2000. 25077268

[59] Office on Smoking and Health (US). The health consequences of involuntary exposure to tobacco smoke: a report of the surgeon general. Centers for Disease Control and Prevention (US). 2006. 20669524

[60] Orton S, Jones LL, Cooper S, Lewis S, Coleman T (2014). Predictors of children’s secondhand smoke exposure at home: a systematic review and narrative synthesis of the evidence. PLoS ONE.

[61] Osborne M, Self-Brown S. Tobacco use and home safety hazards in a sample of child welfare-involved caregivers, monthly research seminar. 2019. Georgia State University, School of Public Health Research Seminar.

[62] Padrón A, Galán I, García-Esquinas E, Fernández E, Ballbè M, Rodríguez-Artalejo F (2016). Exposure to secondhand smoke in the home and mental health in children: a population-based study. Tob Control.

[63] Pizacani BA, Martin DP, Stark MJ, Koepsell TD, Thompson B, Diehr P (2002). Household smoking bans: which households have them and do they work?. Prev Med.

[64] Prochaska J, Diclemente C (1982). (1982) Transtheoretical therapy: toward a more integrative model of change. Psychother Theory Res Pract.

[65] Radey M, & Wilke DJ. The importance of job demands and supports: promoting retention among child welfare workers. Child Adoles Soc Work J. 2021;40:1–13. 10.1007/s10560-021-00762-z PMC8093368 33967382

[66] Self-Brown S, Osborne MC, Lai BS, De Veauuse Brown N, Glasheen TL, Adams MC (2017). Initial findings from a feasibility trial examining the SafeCare Dad to Kids program with marginalized fathers. J Fam Viol.

[67] Self-Brown S, Reuben K, Perry EW, Bullinger LR, Osborne MC, Bielecki J, Whitaker D (2020). The impact of COVID-19 on the delivery of an evidence-based child maltreatment prevention program: understanding the perspectives of SafeCare® Providers. J Fam Viol.

[68] Silovsky JF, Bard D, Chaffin M, Hecht D, Burris L, Owora A, … & Lutzker J. Prevention of child maltreatment in high-risk rural families: a randomized clinical trial with child welfare outcomes. Children Youth Serv Rev. 2011;33(8):1435–1444.

[69] Shonkoff JP, Boyce WT, McEwen BS (2009). Neuroscience, molecular biology, and the childhood roots of health disparities: building a new framework for health promotion and disease prevention. JAMA.

[70] Teresi JA, Yu X, Stewart AL, Hays RD (2022). Guidelines for designing and evaluating feasibility pilot studies. Med Care.

[71] Tsai J, Homa DM, Gentzke AS, Mahoney M, Sharapova SR, Sosnoff CS, … & Trivers KF. Exposure to secondhand smoke among nonsmokers—United States, 1988–2014. Morbid Mortal Wkly Rep. 2018;67(48):1342. 10.15585/mmwr.mm6748a3 PMC6329485 30521502

[72] U.S. Department of Health & Human Services (USDHHS), Administration for Children and Families, Administration on Children, Youth and Families, Children’s Bureau. Child Maltreatment 2020. 2022. https://www.acf.hhs.gov/cb/data-research/child-maltreatment.

[73] Wade R Jr, Cronholm PF, Fein JA, et al. Household and community-level adverse childhood experiences and adult health outcomes in a diverse urban population. Child Abuse Neglect. 2016;52:135–45. 10.1016/j.chiabu.2015.11.021. 10.1016/j.chiabu.2015.11.021 26726759

[74] Wakefield MA, Chaloupka FJ, Kaufman NJ, Orleans CT, Barker DC, Ruel EE (2000). Effect of restrictions on smoking at home, at school, and in public places on teenage smoking: cross sectional study. BMJ.

[75] Walker SP, Chang SM, Vera-Hernández M, Grantham-McGregor S (2011). Early childhood stimulation benefits adult competence and reduces violent behavior. Pediatrics.

[76] Walker SP, Chang SM, Wright A, Osmond C, Grantham-McGregor SM (2015). Early childhood stunting is associated with lower developmental levels in the subsequent generation of children. J Nutr.

[77] Wegman HL, Stetler C (2009). A meta-analytic review of the effects of childhood abuse on medical outcomes in adulthood. Psychosom Med.

[78] Whitaker DJ, Self-Brown S, Hayat MJ, Osborne MC, Weeks EA, Reidy DE, Lyons M (2020). Effect of the SafeCare© intervention on parenting outcomes among parents in child welfare systems: a cluster randomized trial. Preventative Medicine.

[79] Whitehead TP, Metayer C, Wiemels JL, Singer AW, Miller MD (2016). Childhood leukemia and primary prevention. Curr Probl Pediatr Adolesc Health Care.

[80] Williams RS, Stollings JH, Bundy Ł, Haardörfer R, Kreuter MW, Mullen PD, … & Kegler MC. A minimal intervention to promote smoke-free homes among 2–1–1 callers: North Carolina randomized effectiveness trial. PLoS One. 2016;11(11):e0165086. 10.1371/journal.pone.0165086 PMC5091897 27806060

[81] Wyatt Kaminski J, Valle LA, Filene JH, et al. A Meta-analytic Review of Components Associated with Parent Training Program Effectiveness. J Abnorm Child Psychol. 2008;36:567–89. 10.1007/s10802-007-9201-9. 10.1007/s10802-007-9201-9 18205039

[82] Yang MY, Maguire-Jack K (2018). Individual and cumulative risks for child abuse and neglect. Fam Relat.

[83] Zhang X, Martinez-Donate AP, Kuo D, Jones NR, Palmersheim KA (2012). Trends in home smoking bans in the USA, 1995–2007: prevalence, discrepancies and disparities. Tob Control.

[84] Zhou S, Rosenthal DG, Sherman S, Zelikoff J, Gordon T, Weitzman M (2014). Physical, behavioral, and cognitive effects of prenatal tobacco and postnatal secondhand smoke exposure. Curr Probl Pediatr Adolesc Health Care.

