# Supplementary material for: Systematic braiding of Smoke-Free Home SafeCare to address child maltreatment risk and secondhand smoke exposure: findings from a pilot study
Source: Pilot Feasibility Stud. 2023 May 12;9:81. doi: 10.1186/s40814-023-01303-4 (PMC10175921; doi:10.1186/s40814-023-01303-4)
Supplement: Supplementary file 1 — Additional file 1. Smoke Free Home SafeCare – Pilot and Feasibility Study. [file 40814_2023_1303_MOESM1_ESM.docx]

**Smoke Free Home SafeCare – Pilot and Feasibility Study**

**Caregiver participants**

Analyzed (n = 8)

Post-intervention data (n = 8)

Stopped participation during intervention (n = 1)

Allocated to Intervention (n = 10)

Started intervention (n = 9)

Excluded (n = 18 )

No smoking in the home (n = 10)

Parent younger than 18 years old (n = 1)

Eligible, but no contact post-screening (n = 7)

Assessed for Eligibility (n = 28)
