# Supplementary material for: Systematic braiding of Smoke-Free Home SafeCare to address child maltreatment risk and secondhand smoke exposure: findings from a pilot study
Source: Pilot Feasibility Stud. 2023 May 12;9:81. doi: 10.1186/s40814-023-01303-4 (PMC10175921; doi:10.1186/s40814-023-01303-4)
Supplement: Supplementary file 2 — Additional file 2. CONSORT checklist of information to include when reporting a pilot trial*. [file 40814_2023_1303_MOESM2_ESM.docx]

CONSORT checklist of information to include when reporting a pilot trial*

| **Section/topic and item No** | **Standard checklist item** | **Extension for pilot trials** | **Page No where item is**  **reported** |
| --- | --- | --- | --- |
| **Title and abstract** |  |  |  |
| 1a | Identification as a randomised trial in the title | Identification as a pilot or feasibility ~~randomised~~ trial in the title | 1 |
| 1b | Structured summary of trial design, methods, results, and conclusions (for specific guidance see CONSORT for abstracts) | Structured summary of pilot trial design, methods, results, and conclusions (for specific guidance see CONSORT abstract extension for pilot trials) | 2-3 |
| **Introduction** |  |  |  |
| Background and objectives: |  |  |  |
| 2a | Scientific background and explanation of rationale | Scientific background and explanation of rationale for future definitive trial, and reasons for ~~randomised~~ pilot trial | 4-9 |
| 2b | Specific objectives or hypotheses | Specific objectives or research questions for pilot trial | 9 |
| **Methods** |  |  |  |
| Trial design: |  |  |  |
| 3a | Description of trial design (such as parallel, factorial) including allocation ratio | Description of pilot trial design (such as parallel, factorial) including allocation ratio | 9-10,13-14 |
| 3b | Important changes to methods after trial commencement (such as eligibility criteria), with reasons | Important changes to methods after pilot trial commencement (such as eligibility criteria), with reasons | NA |
| Participants: |  | |  |
| 4a | Eligibility criteria for participants | |  |
| 4b | Settings and locations where the data were collected | |  |
| 4c |  | How participants were identified and consented | 10-15 |
| Interventions: |  |  |  |
| 5 | The interventions for each group with sufficient details to allow replication, including how and when they were actually administered |  |  |
| Outcomes: |  |  |  |
| 6a | Completely defined prespecified primary and secondary outcome measures, including how and when they were assessed | Completely defined prespecified assessments or measurements to address each pilot trial objective specified in 2b, including how and when they were assessed | 10-15 |
| 6b | Any changes to trial outcomes after the trial commenced, with reasons | Any changes to pilot trial assessments or measurements after the pilot trial commenced, with reasons | NA |
| 6c |  | If applicable, prespecified criteria used to  judge whether, or how, to proceed with future definitive trial | NA |

| Sample size: |  |  |  |
| --- | --- | --- | --- |
| 7a | How sample size was determined | Rationale for numbers in the pilot trial | 10-11, 14 |
| 7b | When applicable, explanation of any interim analyses and stopping guidelines |  | NA |
| Randomisation: |  |  |  |
| Sequence generation: | | |  |
| 8a | Method used to generate the random allocation sequence |  |  |
| 8b | Type of randomisation; details of any restriction (such as blocking and block size) | Type of randomisation(s); details of any restriction (such as blocking and block size) | NA |
| Allocation concealment mechanism: | | |  |
| 9 | Mechanism used to implement the random allocation sequence (such as sequentially numbered containers), describing any steps taken to conceal the sequence until interventions were assigned |  |  |
| Implementation: | | |  |
| 10 | Who generated the random allocation sequence, enrolled participants, and assigned participants to interventions |  |  |
| Blinding: |  |  |  |
| 11a | If done, who was blinded after assignment to interventions (eg, participants, care providers, those assessing outcomes) and how |  |  |
| 11b | If relevant, description of the similarity of interventions |  |  |
| Analytical methods: | | |  |
| 12a | Statistical methods used to compare groups for primary and secondary outcomes | Methods used to address each pilot trial objective whether qualitative or quantitative | 15-16 |
| 12b | Methods for additional analyses, such as subgroup analyses and adjusted  analyses | Not applicable |  |
| **Results** |  |  |  |
| Participant flow (a diagram is strongly recommended): | | |  |
| 13a | For each group, the numbers of participants who were randomly assigned, received intended treatment, and were analysed for the primary outcome | For each group, the numbers of participants who were approached and/or assessed for eligibility, randomly assigned, received intended treatment, and were assessed for each objective | 10-11, 14 |
| 13b | For each group, losses and exclusions after randomisation, together with reasons |  |  |
| Recruitment: |  |  |  |

| 14a | Dates defining the periods of recruitment and follow-up |  |  |
| --- | --- | --- | --- |
| 14b | Why the trial ended or was stopped | Why the pilot trial ended or was stopped | 10-11, 14 |
| Baseline data: |  |  |  |
| 15 | A table showing baseline demographic and clinical characteristics for each group |  |  |
| Numbers analysed: | | |  |
| 16 | For each group, number of participants (denominator) included in each analysis and whether the analysis was by original assigned groups | For each objective, number of participants (denominator) included in each analysis. If relevant, these numbers should be by randomised group | 16 |
| Outcomes and estimation: | | |  |
| 17a | For each primary and secondary outcome, results for each group, and the estimated effect size and its precision (such as 95% confidence interval) | For each objective, results including expressions of uncertainty (such as 95% confidence interval) for any estimates. If relevant, these results should be by randomised group | NA |
| 17b | For binary outcomes, presentation of both absolute and relative effect sizes is recommended | Not applicable |  |
| Ancillary analyses: | | |  |
| 18 | Results of any other analyses performed, including subgroup analyses and adjusted analyses, distinguishing prespecified from  exploratory | Results of any other analyses performed that could be used to inform the future definitive trial | 16-20 |
| Harms: |  |  |  |
| 19 | All important harms or unintended effects in each group (for specific guidance see CONSORT for harms) |  |  |
| 19a |  | If relevant, other important unintended consequences | NA |
| **Discussion** |  |  |  |
| Limitations: |  |  |  |
| 20 | Trial limitations, addressing sources of potential bias, imprecision, and, if relevant, multiplicity of analyses | Pilot trial limitations, addressing sources of potential bias and remaining uncertainty about feasibility | 21-22, 25 |
| Generalisability: |  |  |  |
| 21 | Generalisability (external validity, applicability) of the trial findings | Generalisability (applicability) of pilot trial methods and findings to future definitive trial and other studies | 26 |
| Interpretation: |  |  |  |
| 22 | Interpretation consistent with results, balancing benefits and harms, and considering other relevant evidence | Interpretation consistent with pilot trial objectives and findings, balancing potential benefits and harms, and  considering other relevant evidence | 20-25 |
| 22a |  | Implications for progression from pilot to future definitive trial, including any proposed amendments | 21, 24-26 |
| **Other information** | | |  |

| Registration: |  |  |  |
| --- | --- | --- | --- |
| 23 | Registration number and name of trial registry | Registration number for pilot trial and name of trial registry | 27 |
| Protocol: |  |  |  |
| 24 | Where the full trial protocol can be accessed, if available | Where the pilot trial protocol can be accessed, if available | 27 |
| Funding: |  |  |  |
| 25 | Sources of funding and other support (such as supply of drugs), role of funders |  |  |
| 26 |  | Ethical approval or approval by research review committee, confirmed with reference number | 11, 27 |
|  |  |  |  |

*Here a pilot trial means any randomised study conducted in preparation for a future definitive RCT, where the main objective of the pilot trial is to assess feasibility.
